# Diastolic Left Ventricular Function in Relation to Urinary and Serum Collagen Biomarkers in a General Population

**DOI:** 10.1371/journal.pone.0167582

**Published:** 2016-12-13

**Authors:** Zhen-Yu Zhang, Susana Ravassa, Wen-Yi Yang, Thibault Petit, Martin Pejchinovski, Petra Zürbig, Begoña López, Fang-Fei Wei, Claudia Pontillo, Lutgarde Thijs, Lotte Jacobs, Arantxa González, Thomas Koeck, Christian Delles, Jens-Uwe Voigt, Peter Verhamme, Tatiana Kuznetsova, Javier Díez, Harald Mischak, Jan A. Staessen

**Affiliations:** 1 Studies Coordinating Centre, Research Unit Hypertension and Cardiovascular Epidemiology, KU Leuven Department of Cardiovascular Diseases, University of Leuven, Leuven, Belgium; 2 Program of Cardiovascular Diseases, Centre for Applied Medical, University of Navarra, Pamplona, Spain; 3 Instituto de Investigación Sanitaria de Navarra, Pamplona, Spain; 4 Mosaiques Diagnostic and Therapeutics AG, Hannover, Germany; 5 BHF Glasgow Cardiovascular Research Centre, University of Glasgow, Glasgow, United Kingdom; 6 Research Unit Cardiology, KU Leuven Department of Cardiovascular Diseases, University of Leuven, Leuven, Belgium; 7 Centre for Molecular and Vascular Biology, KU Leuven Department of Cardiovascular Diseases, University of Leuven, Leuven, Belgium; 8 Department of Cardiology and Cardiac Surgery, University of Navarra Clinic, University of Navarra, Pamplona, Spain; 9 R&D Group VitaK, Maastricht University, Maastricht, The Netherlands; Universita degli Studi di Roma La Sapienza, ITALY

## Abstract

Current knowledge on the pathogenesis of diastolic heart failure predominantly rests on case-control studies involving symptomatic patients with preserved ejection fraction and relying on invasive diagnostic procedures including endomyocardial biopsy. Our objective was to gain insight in serum and urinary biomarkers reflecting collagen turnover and associated with asymptomatic diastolic LV dysfunction. We randomly recruited 782 Flemish (51.3% women; 50.5 years). We assessed diastolic LV function from the early and late diastolic peak velocities of the transmitral blood flow and of the mitral annulus. By sequencing urinary peptides, we identified 70 urinary collagen fragments. In serum, we measured carboxyterminal propeptide of procollagen type 1 (PICP) as marker of collagen I synthesis and tissue inhibitor of matrix metalloproteinase type 1 (TIMP-1), an inhibitor of collagen-degrading enzymes. In multivariable-adjusted analyses with Bonferroni correction, we expressed effect sizes per 1-SD in urinary collagen I (uCI) or collagen III (uCIII) fragments. In relation to uCI fragments, e’ decreased by 0.183 cm/s (95% confidence interval, 0.017 to 0.350; *p* = 0.025), whereas E/e’ increased by 0.210 (0.067 to 0.353; *p* = 0.0012). E/e’ decreased with uCIII by 0.168 (0.021 to 0.316; *p* = 0.018). Based on age-specific echocardiographic criteria, 182 participants (23.3%) had subclinical diastolic LV dysfunction. Partial least squares discriminant analysis contrasting normal *vs*. diastolic LV dysfunction confirmed the aforementioned associations with the uCI and uCIII fragments. PICP and TIMP-1 increased in relation to uCI (*p*<0.0001), whereas these serum markers decreased with uCIII (*p*≤0.0006). Diastolic LV dysfunction was associated with higher levels of TIMP-1 (653 *vs*. 696 ng/mL; *p* = 0.013). In a general population, the non-invasively assessed diastolic LV function correlated inversely with uCI and serum markers of collagen I deposition, but positively with uCIII. These observations generalise previous studies in patients to randomly recruited people, in whom diastolic LV function ranged from normal to subclinical impairment, but did not encompass overt diastolic heart failure.

## Introduction

Diastolic heart failure, also known as heart failure with preserved ejection fraction (HFpEF) represents half of all heart failure cases. It has a prognosis as dire as heart failure with reduced ejection fraction with a rate of cardiovascular mortality of over 30% within one year of the first hospital admission. In patients with diastolic heart failure, the left ventricular (LV) wall undergoes fibrosis characterised by increased interstitial deposition [1] and cross-linking of collagen I at the detriment of collagen III [2,3]. Small increases in the collagen I/III ratio augment myocardial stiffness, thereby reducing early diastolic LV filling and increasing LV filling pressure [4,5]. However, information on the asymptomatic phases of the disease remains scarce. This knowledge gap is particularly relevant, because the prevalence of asymptomatic diastolic LV dysfunction in the general population is as high as 25%[6] with a 10% risk of deterioration over 5 years [7].

Capillary electrophoresis coupled with high-resolution mass spectrometry (CE-MS) enables detection of over 5000 distinct peptide fragments in urine samples [8]. Our previous studies revealed a unique urinary proteomic signature, which in case-control studies [9] and in the general population [10] was reproducibly associated with subclinical diastolic LV dysfunction and which predicted the incidence of adverse cardiovascular outcomes over and beyond traditional cardiovascular risk factors [11]. We hypothesised that jointly linking diastolic LV dysfunction to urinary and serum markers of collagen turnover might increase our understanding of LV dysfunction. With the aim to generalise observations in patients with diastolic heart failure [2–5] to the early still asymptomatic stage of the disease in the population at large, we analysed the Flemish Study on Environment, Genes and Health Outcomes (FLEMENGHO) [10]. First, we searched for association of diastolic LV function with individual urinary peptides with known amino-acid sequence, thereby identifying collagen types as the parent proteins. Next, we correlated single urinary peptides significantly associated with diastolic LV function with circulating markers of cardiac collagen turnover, which in previous studies [5,12] predicted mortality and cardiovascular events.

## Materials and Methods

### Study population

FLEMENGHO complies with the Helsinki declaration [13] for research in human subjects. The Ethics Committee of the University of Leuven approved the study [10]. At each contact, participants gave informed written consent. Recruitment for FLEMENGHO started in 1985 [10,14–17]. From August 1985 until November 1990, a random sample of the households living in a geographically defined area of Northern Belgium was investigated with the goal to recruit an equal number of participants in each of six subgroups by sex and age (20–39, 40–59, and ≥60 years). All household members with a minimum age of 20 years were invited to take part, provided that the quota of their sex-age group had not yet been satisfied. From June 1996 until January 2004 recruitment of families continued using the former participants (1985−1990) as index persons and also including teenagers. The initial participation rate was 78·0%. The participants were repeatedly followed up at the field centre in the catchment area (North Limburg, Belgium). From May 2005 until May 2010, an invitation letter was mailed to 1208 former participants for a follow-up examination. However, 153 were unavailable, because they had died (n = 26), had been institutionalised or were too ill (n = 27), or had moved out of the area (n = 100). Of the remaining 1055 former participants, 828 renewed informed consent [10]. The participation rate at re-examination was 78.5%. We excluded 46 participants from analysis, because serum samples were unavailable (n = 22), or because arrhythmia (n = 8), paced heart rhythm (n = 3) or poor echocardiographic image quality (n = 13) rendered assessment of diastolic LV function difficult. Thus, the number of participants statistically analysed totalled 782.

### Echocardiography

The acquisition, offline analysis, staging of diastolic LV function and quality control are extensively described in previous publications [6,7,18] and summarised below.

#### Data acquisition

One observer (TKu) did the ultrasound examination [6], using a Vivid7 Pro (GE Vingmed, Horten, Norway) device interfaced with a 2.5- to 3.5-MHz phased-array probe. For off-line analysis, she recorded at least five heart cycles according to the recommendations of the American Society of Echocardiography [19]. M-mode echocardiograms of the left ventricle (LV) were recorded from the parasternal long-axis under control of the two-dimensional image. The ultrasound beam was positioned just below the mitral valve at the level of the posterior tendinous chords. To record the mitral and pulmonary vein (PV) flow velocities from the apical window, the observer positioned the Doppler sample volume at the mitral valve tips, in the right superior PV, and between the LV outflow and mitral inflow, respectively. From the apical window, the observer placed a 5-mm Doppler sample at the septal, lateral, inferior and posterior sites of the mitral annulus to record low-velocity, high-intensity myocardial signals at a high frame rate (>190 frames per second), while ensuring parallel alignment of the ultrasound beam with the myocardial segment of interest.

#### Off-line analysis

One reader (TKu) analysed the digitally stored images, averaging three heart cycles, using a workstation running EchoPac software, version 4.0.4 (GE Vingmed, Horten, Norway). LV internal diameter and interventricular septal and posterior wall thickness were measured at end-diastole from the 2-dimensionally guided M-mode tracing. When optimal orientation of M-mode ultrasound beam could not be obtained, the reader performed linear measurements on correctly oriented 2-dimensional images. End-diastolic LV dimensions were used to calculate LV mass [19]. Left atrial (LA) volume was calculated using the prolate-elipsoid method from the LA dimensions in three orthogonal planes and indexed to body surface area [19]. From the transmitral flow signal, the reader determined peak early diastolic velocity (E), peak late diastolic velocity (A), the E/A ratio, and the transmitral A flow duration. From the PV flow signal, she measured the duration of PV reversal flow during atrial systole. From the tissue Doppler recordings, the observer measured peak early (e') and peak late (a') diastolic mitral annular velocities, and the e'/a' ratio at the four acquisition sites (septal, lateral, inferior, and posterior).

#### Staging of diastolic LV function

**Fig 1:**
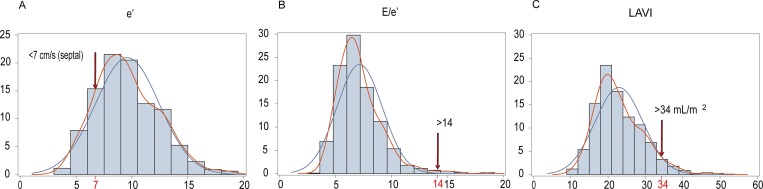
**Distributions of: (A) the peak velocity of the mitral annular movement during early diastole (e’); (B) the ratio of the peak velocities of transmitral blood flow to mitral annular movement in early diastole; and (C) the left atrial volume index (LAVI).** In line with the US guideline (J Am Soc Echocardiogr 2016;29:277–314), e’ was measured at septal wall in panel A, but was the average of the septal and lateral sample sites in panel B. Arrows indicate the cut-off values for diastolic LV dysfunction recommended for clinical use in patients with advanced diastolic dysfunction.

S1 Table summarises the physiological interpretation of the echocardiographic measurements reflecting diastolic LV function. As shown in Fig 1, guideline-driven echocardiographic criteria to stage patients with advanced diastolic LV dysfunction [20] leave a large proportion of people unclassified in population studies [6,21]. We therefore developed age-specific criteria in a healthy reference sample drawn from FLEMENGHO [6] and replicated these criteria in a European population study [21]. To stage diastolic LV function in our current study, as previously described [6,21], we combined the velocities of the transmitral blood flow and the mitral annular movement. Group 1 included patients with an abnormally low age-specific transmitral E/A ratio indicative of impaired relaxation, but without evidence of increased LV filling pressures (E/e' ≤ 8.5). Group 2 had mildly-to-moderately elevated LV filling pressure (E/e' > 8.5) and an E/A ratio within the normal age-specific range. Differences in durations between the transmitral A flow (Ad) and the reverse flow in the pulmonary veins (ARd) during atrial systole (Ad < ARd + 10) and the left atrial volume index (≥ 29 mL/m2; the 97.5th percentile of the distribution in a reference sample of 239 healthy FLEMENGHO participants [6]) were checked to confirm elevation of LV filling pressure (S1 Table). Group 3 had an elevated E/e' ratio and an abnormally low age-specific E/A ratio (combined dysfunction). We combined these three groups for analysis.

#### Quality control

Intra-observer reproducibility was the 2-SD interval about the mean of the relative differences across pairwise readings. The intra-observer reproducibility for the tissue Doppler peak velocities across the four sampling sites ranged from 4.5% to 5.3% for e’ and from 4.0% to 4.5% for a’ [6]. Reproducibility was 2.2% for internal end-diastolic LV diameter, 4.6% for LV wall thickness, and 4.3% for LV mass [18].

### Urinary peptides

Participants collected 24-h urine samples within one week of the echocardiographic examinations. Aliquots (0.7 mL) were stored at –80°C and thawed immediately before analysis. Previous publications provide a detailed account of the methods applied for preparation of the urine samples and running the CE-MS analysis [22,23]. For targeted sequencing, urine samples were analysed on a Dionex Ultimate 3000 RSLS nano flow system (Dionex, Camberly, UK) or on a Beckman CE, coupled to an Orbitrap Velos MS instrument (Thermo Scientific, Waltham, Massachusetts, US) [24]. The data files were analysed using Proteome Discoverer 1.2 (precursor mass tolerance, 10 ppm; fragment mass tolerance, 0.05 Da) and were searched against the UniProt human non-redundant database without enzyme specificity. No fixed modifications were selected. Oxidation of methionine and proline were considered as variable modifications. The criteria for accepting sequences were high confidence (Xcorr ≥ 1.9) and absence of unmodified cysteine. A strong correlation between peptide charge at the working pH of 2 and capillary electrophoresis migration time was used to avoid falsely characterised peptides [25].

We identified 88 sequenced peptides with mean signal amplitude different from undetectable in over 95% of participants. Proteomics and peptidomics data usually display missing values, which result from either the absence of a particular peptide in certain datasets or from the fact that this particular peptide may be below of the limit of detection of the technological platform. For the current analysis, the values of peptides undetectable in less than 5% of randomly varying study participants were set to the minimum of the distribution of each sequenced peptide. This strategy has been validated by other research groups [26,27]. Furthermore, the cardiac extracellular matrix (ECM) is predominantly composed of fibrillar collagen I (85%) and III (11%) and small amounts of collagen IV and V co-distributed with collagen I. We therefore confined our analyses to 70 urinary peptide fragments, which could be traced back to the aforementioned collagen types (S2 Table).

### Circulating biomarkers

On the day of echocardiography, venous blood samples were drawn after at least 8 hours of fasting. Serum was analysed for carboxyterminal propeptide of procollagen I (PICP), a marker of collagen I synthesis; carboxyterminal telopeptide of collagen I (CITP) and tissue inhibitor of the matrix metalloproteinase type 1 (TIMP-1) as markers of degradation of collagen I; and amino terminal propeptide of procollagen type III (PIIINP), a marker of synthesis and degradation of collagen III (S2 Fig). Serum markers were measured in 740 of 782 participants (94.6%). As described previously [3], PICP (Quidel Corporation, San Diego, CA), TIMP-1 (GE Healthcare Life Sciences, Buckinghamshire, UK) and PIIINP (MyBioSource, San Diego, CA) were quantified by sandwich enzyme linked immunosorbent assay and CITP by a quantitative enzyme immunoassay (Orion Diagnostica, Espoo, Finland). The detection limits were 0.2 μg/L for PICP (inter- and intra-assay coefficients of variability, 6.4% and 4.5%), 0.3 μg/L for CITP (13.1% and 10.0%), 1.25 ng/mL for TIMP-1 (12.8% and 2.6%), and 31.3 pg/mL for PIIINP (<15.0%) [3].

### Other measurements

Blood pressure was the average of five consecutive auscultatory readings obtained according to European guidelines [28] with a standard mercury sphygmomanometer with the participant resting in the seated position for at least 10 minutes. Hypertension was a blood pressure of at least 140 mm Hg systolic or 90 mm Hg diastolic or use of antihypertensive drugs. Body mass index was weight in kilograms divided by height in meters squared. Plasma glucose and serum total and high-density lipoprotein (HDL) cholesterol, creatinine, γ-glutamyltransferase (biomarker of alcohol intake) and insulin were measured by automated methods in a single certified laboratory. Glomerular filtration rate was derived by the Chronic Kidney Disease Epidemiology Collaboration equation [29]. We graded chronic kidney disease according to the National Kidney Foundation K/DOQI guideline [30] into stages 1, 2, 3A, 3B, 4 and 5 based on eGFR values of ≥90, 60–89, 45–59, 30–44, 15–29 and <15 mL/min/1.73 m2. Diabetes mellitus was a self-reported diagnosis, a fasting glucose level of 7 mmol/L or higher, or use of antidiabetic agents [31].

### Statistical Analysis

For database management and statistical analysis, we used the SAS system, version 9.4 (SAS Institute Inc., Cary, NC). Means were compared using the large-sample z-test and proportions by Fisher’s exact test. We normalised the distributions of γ-glutamyltransferase, insulin and PIIINP by a logarithmic transformation.

We identified covariables to be retained in the analyses by a stepwise regression procedure with *p*-values for covariables to enter and stay in the models set at 0.15. In continuous analyses, we standardised diastolic LV function for the average in the whole study population (mean or ratio) of the covariables identified by stepwise regression. While accounting for covariables, we regressed the indexes of diastolic LV function on the urinary peptide markers and constructed–log10 probability plots. Based on the number of parent collagen proteins (types I, III, IV and V), we adjusted significance levels by the Bonferroni method. In the next step of our analyses, we applied partial least squares discriminant analysis (PLS-DA). PLS-DA is a statistical technique that constructs predictive models for categorical outcomes in relation to correlated high dimensional predictors. PLS-DA allowed us to identify a set of independent latent factors that were linear combinations of the urinary peptides and that maximised the covariance between the urinary peptides and the variables describing diastolic LV function. We studied normal *vs*. diastolic dysfunction in relation to the latent factors. We retained the smallest number of latent factors for which the predicted residual sums of squares (PRESS, calculated using leave-one-out cross-validation) did not differ significantly (*p*>0.10) from the model with the minimum PRESS value as assessed by the van der Voet T2 statistic. The importance of each urinary peptide in the construction of the PLS factors and in the association of diastolic function was assessed from the Variable Importance in Projection (VIP) scores of Wold. Using principal component analysis, we summarized the urinary peptides with a VIP score higher than 1.0 and a correlation coefficient of less than –0.04 or higher than 0.04 into a single factor. Finally, we constructed Receiver Operating Characteristic (ROC) plots and evaluated the area under the ROC curve (AUC).

## Results

### Characteristics of participants

Of 782 participants, 401 (51.3%) were women. All were White Europeans. Mean values (SD) in the 782 participants were 50.5 (15.5) years for age, 26.4 (4.2) kg/m2 for body mass index, 129.1 (17.5) mm Hg and 79.6 (9.5) mm Hg for systolic and diastolic blood pressure, and 5.25 (0.97) mmol/L for total cholesterol. Among all participants, 327 (41.8%) had hypertension, of whom 199 (60.9%) were on antihypertensive drug treatment, and 9 (1.2%) had diabetes.

The prevalence of diastolic LV dysfunction amounted to 182 (23.3%). Two participants had tricuspid regurgitation and 10 had an E/e’ ratio exceeding 14. Among participants with normal or impaired diastolic LV function only two and seven had an ejection fraction of less than 50%. There was no difference in the ejection fraction between these two groups (68.2 *vs*. 69.0%, *p* = 0.29). Table 1 shows that cardiovascular risk factors were more prevalent or elevated in participants with diastolic dysfunction compared with those with normal LV function, with exception of the proportion of women, smokers and patients with diabetes. Fig 1 shows the distributions of echocardiographic indexes, e’ and E/e’. Diastolic LV dysfunction was characterised by impaired relaxation in 68 patients (37.4%) or an elevated filling pressure in the presence of a normal (90 [49.5%]) or low (24 [13.2%]) age-specific E/A ratio [6,21]. The echocardiographic characteristics of participants by these categories of diastolic LV function appear in Table 2. Compared with participants with normal LV function, patients with diastolic LV dysfunction more frequently (*p*≤0.003) used medications: any diuretic (5.3% *vs*. 25.3%), loop diuretics (0.2% *vs*. 2.2%), spironolactone (1.0% *vs*. 7.7%), β-blockers (9.0% *vs*. 36.3%), angiotensin-converting enzyme inhibitors (3.0% *vs*. 9.3%), angiotensin I type-1 receptor blockers (2.2% *vs*. 10.4%) and vasodilators including calcium-channel blockers and α-blockers (2.8% *vs*. 11.0%).

**Table 1:** Characteristics of 782 Participants by Category of the Diastolic LV Function

Characteristic	Normal	Dysfunction	*p*
Number in category	600	182	
Number of subjects (%)			
Women	298 (49.7)	103 (56.6)	0.10
Smokers	129 (21.5)	29 (15.9)	0.10
Drinking alcohol	435 (72.5)	107 (58.8)	0.0004
Hypertension	180 (30.0)	147 (80.8)	<0.0001
Antihypertensive treatment	97 (53.9)	102 (69.4)	<0.0001
Diabetes mellitus	4 (0.7)	5 (2.8)	0.10
Mean (SD) of characteristic			
Age (years)	46.4 (13.8)	64.2 (12.7)	<0.0001
Body mass index (kg/m2)	25.8 (4.0)	28.6 (4.4)	<0.0001
Waist-to-hip ratio	0.86 (0.08)	0.90 (0.08)	<0.0001
Office blood pressure (mmHg)			
Systolic pressure	125.1 (15.2)	142.2 (18.2)	<0.0001
Diastolic pressure	79.0 (9.3)	81.5 (10.1)	0.0021
Mean arterial pressure	94.3 (10.2)	101.7 (10.3)	<0.0001
Heart rate (beats per minute)	60.3 (9.2)	62.1 (11.2)	0.053
Biochemical data			
Serum creatinine (μmol/L)	82.5 (13.5)	86.9 (20.2)	0.0072
eGFR (mL/min/1.73 m2)	83.2 (16.1)	72.4 (14.6)	<0.0001
Total cholesterol (mmol/L)	5.17 (0.96)	5.51 (0.98)	<0.0001
HDL cholesterol (mmol/L)	1.44 (0.35)	1.38 (0.35)	0.059
Total-to-HDL cholesterol ratio	3.75 (1.01)	4.17 (1.05)	<0.0001
Plasma glucose (mmol/L)	4.84 (0.55)	5.25 (1.25)	<0.0001
γ-Glutamyltransferase (units/L)	22 (12–53)	26 (13–51)	0.013
Insulin (pmol/L)	31.0 (13.9–69.5)	40.9 (21.0–89.6)	<0.0001

eGFR indicates estimated glomerular filtration rate derived by the Chronic Kidney Disease Epidemiology Collaboration equation formula. Office blood pressure was the average of five consecutive readings. Hypertension was an office blood pressure of ≥140 mmHg systolic, or ≥90 mm Hg diastolic, or use of antihypertensive drugs. For γ−glutamyltransferase and insulin reported values are geometric means (interquartile range). Diabetes mellitus was a self-reported diagnosis, a fasting glucose level of ≥7 mmol/L, or use of antidiabetic agents.

**Table 2:** Echocardiographic measurements by category of diastolic LV function

Characteristic	Normal (n = 600)	Dysfunction (n = 182)
Conventional echocardiography		
Left atrial volume, mL	40.9 (12.5)	49.3 (15.4)*
Left atrial volume index, mL/m2	21.8 (5.51)	26.7 (7.59)*
Left ventricular mass, g	165.5 (44.7)	194.3 (55.7)*
Left ventricular mass index, g/m2	88.4 (19.0)	104.9 (25.7)*
Doppler data		
Deceleration time, ms	159.8 (30.6)	189.2 (45.2)*
Isovolumetric relaxation time, ms	94.7 (14.1)	106.8 (18.0)*
E peak, cm/s	77.7 (14.9)	69.0 (17.3)*
A peak, cm/s	59.1 (13.9)	82.1 (15.8)*
E/A ratio	1.40 (0.46)	0.86 (0.25)*
e’ peak, cm/s	12.6 (3.26)	7.77 (1.89)*
a’ peak, cm/s	9.75 (2.07)	11.1 (1.94)*
e’/a’ ratio	1.42 (0.65)	0.73 (0.26)*
E/e’ ratio	6.38 (1.33)	9.26 (2.78)*

* An asterisk indicates a significant difference with normal.

S3 Table lists the covariables considered for entry and retained for multivariable adjustment of diastolic LV function. Based on the results of the stepwise regression analysis, we standardised all echocardiographic indexes of diastolic function for sex, age, body mass index, mean arterial pressure, heart rate, serum total cholesterol, γ−glutamyltransferase and creatinine, plasma glucose, LV mass index and treatment with diuretics, β−blockers and inhibitors of the renin-angiotensin system.

### Urinary biomarkers

In general, urinary peptides derived from collagen I correlated with one another. For instance, the correlation coefficients relating p70635 to the other collagen I fragments ranged from 0.14 to 0.30 (*p*<0.0001 for all). The correlation between the two collagen III fragments, p107460 and p112106, was 0.19 (*p*<0.0001). There was a weak inverse correlation (r = –0.10; *p* = 0.0054) between p70635 (collagen I fragment) and p107460 (collagen III fragment).

Among all 782 participants, eGFR averaged 80.7 (16.4) mL/min/1.73 m2. The prevalence of renal function stages 1, 2, 3A, 3B, 4 and 5 amounted to 175 (22.4%), 552 (70.8%), 49 (6.3%), 5 (0.6%), 1 (0.1%) and 0, respectively. With adjustments applied for mean arterial pressure, waist-to-hip ratio, smoking, γ-glutamyltransferase, the total-to-HDL cholesterol ratio, plasma glucose, and use of antihypertensive medications by drug class, none of the associations of eGFR with the urinary collagen fragments reached significance (*p*≥0.082).

### Continuous analysis

S1 Fig. shows the–log10(*p*) probability plot of the multivariable-adjusted associations of various indexes of diastolic LV function with the urinary peptides. With Bonferroni correction applied, the urinary peptides that remained significantly associated with the Doppler indexes of diastolic LV function included six fragments of collagen I (p70635, p72896, p73697, p77018, p77952 and p115491) and two fragments of collagen III (p107460 and p112106). Focusing on collagen I (Table 3), e’ peak velocity and the e’/a’ ratio decreased respectively with p70635 (effect size per 1-SD increment, –0.183; *p* = 0.025) and p77952 (–0.041; *p* = 0.006), whereas the E/e’ ratio increased with p72896 (0.164; *p* = 0.020), p77018 (0.210; *p* = 0.0012) and p115491 (0.162; *p* = 0.019). The a’ peak velocity declined with p72896 (–0.192; *p* = 0.0024) and p73697 (–0.160; *p* = 0.016). In relation to collagen III fragments, A peak velocity (–1.450; *p* = 0.0024) and the E/e’ ratio (–0.168; *p* = 0.018) declined with p107460 and the A peak also with p112106 (–1.334; *p* = 0.006). Sensitivity analyses with additional adjustment for HDL cholesterol and insulin produced confirmatory results (Table 3). None of the indexes of diastolic LV function was significantly associated with the sequenced collagen IV or V fragments (S2 Table).

**Table 3:** Multivariable-adjusted associations of tissue Doppler indexes with urinary peptides

Urinary peptides (SD)	Collagen Type	Model 1	*p*	Model 2	*p*
		Estimate (95% CI)		Estimate (95% CI)	
A peak					
p107460 (863)	III	–1.450 (–2.502 to –0.398)	0.0024	–1.453 (–2.507 to –0.398)	0.0024
p112106 (3149)	III	–1.334 (–2.378 to –0.289)	0.006	–1.349 (–2.401 to –0.296)	0.0056
e’ peak					
p70635 (728)	I	–0.183 (–0.350 to –0.017)	0.025	–0.170 (–0.335 to –0.005)	0.041
a’ peak					
p72896 (389)	I	–0.192 (–0.330 to –0.053)	0.0024	–0.193 (–0.332 to –0.055)	0.002
p73697 (521)	I	–0.160 (–0.298 to –0.022)	0.016	–0.163 (–0.300 to –0.025)	0.013
e’/a’ peak					
p77952 (1518)	I	–0.041 (–0.072 to –0.009)	0.006	–0.038 (–0.070 to –0.006)	0.011
E/e’					
p72896 (289)	I	0.164 (0.018 to 0.310)	0.020	0.163 (0.017 to 0.309)	0.022
p77018 (1504)	I	0.210 (0.067 to 0.353)	0.0012	0.208 (0.065 to 0.351)	0.0012
p107460 (863)	III	–0.168 (–0.316 to –0.021)	0.018	–0.164 (–0.312 to –0.016)	0.023
p115491 (2362)	I	0.162 (0.019 to 0.305)	0.019	0.161 (0.018 to 0.304)	0.020

Abbreviation: CI, confidence interval. All estimates were adjusted for sex, age, body mass index, mean arterial pressure, heart rate, serum total cholesterol, γ−glutamyltransferase and creatinine, plasma glucose, LVMI and treatment with diuretics, β−blockers and inhibitors of the renin-angiotensin system. Model 2 was additionally adjusted for HDL cholesterol and insulin. Estimates express the change in the dependent variable for 1-SD increase (given between parentheses) in the urinary peptide. *P*-values are Bonferroni adjusted.

#### Categorical analysis

We dichotomised the study population in 600 participants with normal LV function and 182 with diastolic LV dysfunction. The PLS-DA procedure yielded two latent factors accounting for 10.2% and 6.3% of the variance in the urinary peptides and 16.5% in total.

**Fig 2:**
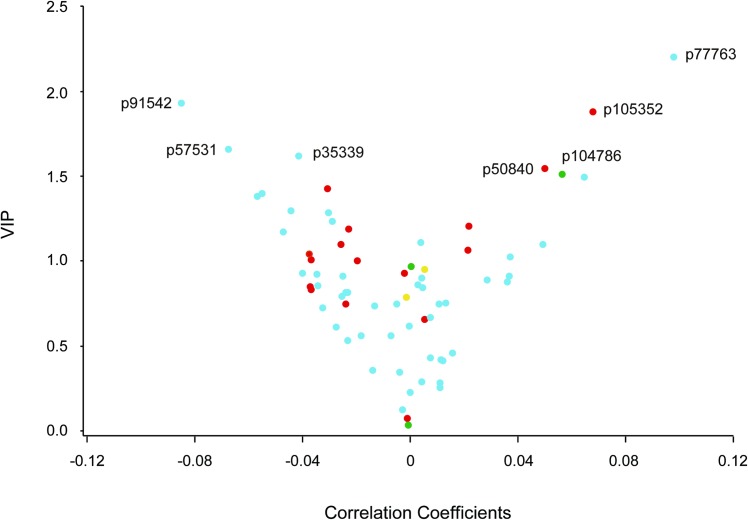
V-plots generated for the PLS DA derived VIP scores versus the centred and rescaled correlation coefficients. We dichotomised the study population in 600 participants with normal LV function and 182 with subclinical diastolic LV dysfunction. VIP is the importance of each urinary fragments in the construction of the PLS factors. The correlation coefficients reflect the association of diastolic LV dysfunction with the urinary collagen fragments. Fragments associated with normal and diastolic LV dysfunction were sitting on the left and right arms, respectively (see Table 3). Fragments derived from collagen I, III, IV and V are labelled blue, red, yellow and green, respectively.

Using a VIP score of 1.5 and a correlation coefficient of –0.04 as cut-offs, normal diastolic LV function (Fig 2, left top side of the V-plot) was associated with the collagen I fragments p35339, p57531, and p91542. Using a VIP score of 1.5 and a correlation coefficient of 0.04 as cut-offs, diastolic dysfunction (Fig 2, right top side of the V-plot) was associated with collagen I fragment p77763, collagen III fragments p50840 and p105352, and collagen V fragment p104786. p77763 is a collagen I fragment with an amino-acid sequence very similar to p77018 (one proline residue being replaced by hydroxyproline; S2 Table), which was related to the indexes of diastolic LV function in the continuous analyses (Table 3 and S1 Fig). In general, the sequences of collagen I fragments associated with normal diastolic function were shorter than those associated with dysfunction (S2 Table). The level of the collagen I fragment p77018 was higher in patients with diastolic LV dysfunction than in participants with normal diastolic function, whereas the opposite was true for the collagen III fragment p107460 (Table 4).

**Table 4:** Serum and urinary biomarkers by category of diastolic LV function

Biomarkers	Unadjusted			Adjusted		
	Normal	Dysfunction	*p*	Normal	Dysfunction	*p*
Urine	n = 600	n = 182		n = 600	n = 182	
p77018 (I)	2751 (56)	3162 (136)	0.0056	2770 (63)	3099 (121)	0.020
p107460 (III)	1788 (35)	1515 (63)	0.0002	1767 (35)	1585 (68)	0.022
p77018/p107460 (I/III)	2.40 (0.19)	4.15 (0.68)	0.015	2.49 (0.25)	3.87 (0.47)	0.0013
Serum	n = 565	n = 175		n = 565	n = 175	
PICP, μg/L	103.7 (1.9)	90.8 (2.8)	0.0002	100.1 (1.8)	102.7 (3.6)	0.54
CITP, μg/L	5.41 (0.08)	6.05 (0.19)	0.003	5.34 (0.09)	6.26 (0.19)	<0.0001
TIMP1, ng/mL	634 (7)	755 (16)	<0.0001	653 (7)	696 (15)	0.013
PIIINP, pg/mL	457 (175–1160)	513 (180–1355)	0.15	447 (175–1160)	562 (180–1355)	0.020

Abbreviations: PICP, carboxyterminal propeptide of procollagen I; CITP, carboxyterminal telopeptide of collagen I; TIMP-1, tissue inhibitor of the matrix metalloproteinase type 1; PIIINP, aminoterminal propeptide of procollagen III. Values are arithmetic mean (SE) or geometric mean (interquartile range). Adjustments included body mass index, serum total cholesterol, γ−glutamyltransferase and creatinine, plasma glucose, and treatment with diuretics, β−blockers and inhibitors of the renin-angiotensin system.

To evaluate diagnostic accuracy, we combined the urinary peptides with a VIP score higher than 1.5 and a correlation coefficient lower than –0.04 or higher than 0.04 into a single factor (p35339, p50840, p57531, p77763, p91542, p104786, p105352), using principal component analysis. The AUC for diagnosing diastolic LV dysfunction was 0.92 (95% CI, 0.89–0.94; *p*<0.0001) yielding a sensitivity, specificity and positive and negative predictive values of 67.6%, 93.3%, 75.5% and 90.5%, respectively.

### Circulating biomarkers

With adjustments applied for metabolic confounders (body mass index, serum total cholesterol, γ-glutamyltransferase and creatinine, and plasma glucose) and concurrent treatment by drug class (Table 4), serum levels of CITP (6.26 *vs*. 5.34 μg/L), TIMP-1 (696 *vs*. 653 ng/mL) and PIIINP (562 *vs*. 447 pg/mL) were significantly higher (*p*≤0.020) in patients with diastolic LV dysfunction (n = 175) than in people with normal function (n = 565), with no between-group differences in serum PICP.

Table 5 lists the associations of circulating collagen biomarkers with the urinary collagen I and III fragments that reached a significance level of 1% or less. With association sizes expressed per 1-SD increment in collagen I fragments, PICP, CITP and PIIINP increased (*p*≤0.0016) by 14.9 μg/L, 0.31 μg/L and 4.63% in relation to p73697. TIMP-1 increased by 21.4 ng/mL (*p* = 0.0013) and by 39.4 ng/mL (*p*<0.0001) in relation to p77018 and p77763, respectively. The association sizes of PICP, CITP and TIMP-1 with collagen III fragments amounted to –5.39 μg/L, –0.62 μg/L and –30.9 ng/mL for p107460 (*p*≤0.0006) and to –0.44 μg/L for CITP.

**Table 5:** Association of urinary collagen fragments with serum biomarkers of collagen turnover

Urinary marker (SD)	Collagen I		Urinary marker (SD)	Collagen III	
Serum markers	Estimate (95% CI)	*p*	Serum markers	Estimate (95% CI)	*p*
p73697 (521)			p107460 (863)		
PICP, μg/L	14.9 (12.0 to 17.8)	<0.0001	PICP, μg/L	–5.39 (–8.47 to –2.31)	0.0006
CITP, μg/L	0.31 (0.16 to 0.47)	<0.0001	CITP, μg/L	–0.62 (–0.76 to –0.47)	<0.0001
PIIINP, %	4.63 (1.77 to 7.50)	0.0016	TIMP-1, ng/mL	–30.9 (–43.9 to –18.0)	<0.0001
p77018 (1504)			p112106 (3149)		
TIMP-1, ng/mL	21.4 (8.36 to 34.5)	0.0013	CITP, μg/L	–0.44 (–0.59 to –0.29)	<0.0001
p77763 (991)					
TIMP-1, ng/mL	39.4 (26.5 to 52.2)	<0.0001			

Abbreviations: CI, confidence interval; PICP, carboxyterminal propeptide of procollagen I; CITP, carboxyterminal telopeptide of collagen I; TIMP-1, tissue inhibitor of the matrix metalloproteinase type 1; PIIINP, aminoterminal propeptide of procollagen type III. Estimates express the change in the serum biomarkers per 1-SD increase in urinary collagen fragments. The urinary peptides were identified in the continuous analyses (S1 Fig and Table 3) with the exception of p77763, which was a marker of diastolic LV dysfunction in the PLS-DA analysis (Fig 2) and had an amino-acid sequence very similar to that of p77018 (one proline residue being replaced by hydroxyproline; S2 Table).

## Discussion

The novel findings in our article, all obtained in a general population, can be summarised as follows: (i) the correlations between urinary collagen I and III fragments were inverse; (ii) in continuous analyses, e’ peak and e’/a’ decreased and E/e’ increased with urinary collagen I fragments, whereas the A peak and E/e’ decreased with urinary collagen III fragments (S1 Fig and Table 3); (iii) the PLS-DA analysis contrasting normal *vs*. diastolic LV dysfunction confirmed the associations with urinary collagen I and III fragments (Fig 2); (iv) PICP, CITP and TIMP-1 increased in relation to urinary collagen I fragments, whereas these serum markers decreased in relation to urinary collagen III (Table 5); and (v) in categorical analyses, diastolic LV dysfunction was associated with higher levels of urinary collagen I fragments, lower levels of urinary collagen III degradation products, and higher levels of CITP and TIMP-1, but not PICP (Table 4).

Collagen I is a stiff fibrillar protein providing tensile strength, whereas collagen III forms an elastic network storing kinetic energy that is released during elastic recoil [32]. Histopathological [33–35] and expression [34] studies of endomyocardial biopsies suggested that in humans chronic heart failure with preserved ejection fraction is characterised by myocardial fibrosis with a predominant increase in collagen I. Zile and colleagues measured myocardial stiffness directly in myocardial biopsies of 70 patients undergoing coronary artery bypass grafting [36]. In comparison with controls without comorbidity and controls with hypertension, patients with hypertension and diastolic heart failure had an increased end-diastolic LV pressure, left atrial volume, and collagen-dependent passive stiffness [36]. In keeping with experimental studies in rats [37], among patients with dilated cardiomyopathy [32], tissue samples of patients with heart failure compared with those from controls with mild global LV dysfunction had a 2- to 6-fold increase in collagen I mRNA, a 2-fold increase in collagen III mRNA, resulting in a higher collagen I/III expression ratio (8.6 *vs*. 6.4). Our current findings are novel, because they show in a general population that echocardiographic indexes of diastolic LV function are associated with sequenced urinary collagen I and III fragments (S1 Fig and Table 3). In addition, the I/III ratio of urinary collagen fragments was higher in patients with diastolic LV dysfunction compared to participants with normal LV function (Table 4). Thus, sequencing of the urinary peptide fragments allowed us to translate previous observations in endomyocardial biopsies [32–35] to people randomly recruited from the general population, in whom diastolic LV function ranged from normal to subclinical dysfunction, but did not encompass overt diastolic heart failure.

During the cardiac cycle, the left atrium acts as a reservoir, receiving pulmonary venous return during LV systole; as a conduit, passively transferring blood to the LV during early diastole; and as a pump, actively priming the LV in late diastole [38]. Stiffening of the LV requires a greater contribution of the atrial contraction to late diastolic LV filling and is associated with a higher a’ peak velocity. Next, as diastolic LV function deteriorates, the a’ peak velocity decreases [39]. This so-called pseudo-normalisation (moderate diastolic LV dysfunction in S1 Table) might therefore underlie the inverse association between a’ peak velocity and urinary collagen I fragments, as observed in our current study (Table 3). However, excluding 13 patients with an e’/a’ ratio higher than unity or all patients with an E/e’ ratio exceeding 8.5 [6] did not confirm this interpretation. An alternative explanation is that worsening of diastolic LV function leads to collagen deposition in the atria [40] with higher collagen I/III ratio [40], impairment of the atrial reservoir function [41], increase in the left atrial volume [36], deterioration of electromechanical coupling [40], and therefore to lower a’ peak velocity.

PICP is released in a 1:1 stoichiometric ratio during conversion of procollagen I to collagen I (S2 Fig) and therefore its serum concentration is a direct indicator of concurrent collagen I synthesis [42]. In patients with hypertensive heart disease [43], circulating PICP, at least in part, originates from the heart, because there is a positive concentration gradient from the coronary sinus towards the antecubital vein with a high correlation between coronary and peripheral levels. This association was not present in normotensive controls [42]. Moreover, serum PICP concentration correlates well with histologically proven myocardial collagen type I deposition [44] and in response to pharmacological intervention changes in serum PICP associate with changes in myocardial collagen type I deposition [45]. Our current study moves the field forward by showing that in the general population PICP increased in relation to p73697, a urinary collagen I fragment, but decreased in relation to p107460, a marker of the more elastic [32] collagen III (Table 5).

Metalloproteinases catalyse the degradation of collagen I resulting in the release of CITP in a 1:1 stoichiometric ratio (S2 Fig). The role of CITP as a reliable biomarker of collagen breakdown is not firmly established, because its association with myocardial fibrosis was inconsistently reported as negative [46] or positive [47]. Notwithstanding the uncertainty in the clinical interpretation of circulating CITP levels, our study (Table 5) revealed that serum CITP correlated positively with p73697, a urinary collagen I fragment associated with worse diastolic LV function, and inversely with p107460, a urinary marker of collagen III breakdown associated with more performant diastolic LV function.

Circulating TIMP-1 inhibits the metalloproteinases and is a pro-fibrotic stimulus. Similar to PICP [43], a positive gradient and a direct correlation exist between the TIMP-1 concentrations in coronary sinus and antecubital vein blood in patients with hypertensive heart disease, but not in normotensive controls [33]. In hypertensive patients with heart failure but normal ejection fraction, elevated estimated capillary wedge pressure compared with normal LV filling pressure was associated with higher TIMP-1 levels and a lower metalloproteinase-1 to TIMP-1 ratio, indicative of lower breakdown of collagen [48]. Zile and coworkers confirmed that in patients with hypertension with or without diastolic heart failure, circulating TIMP-1 levels, but not metalloproteinases, were elevated compared to normotensive controls [36]. Our study moves current knowledge forward by demonstrating that in a general population diastolic LV dysfunction was associated with higher levels of TIMP-1 (Table 4) and that TIMP-1 increased in relation to urinary collagen I fragments (Table 5). By linking circulating TIMP-1 to urinary collagen I fragments, our observation support the hypothesis that an excess of TIMP-1 inhibits collagen degradation, thereby promoting collagen deposition in the myocardium and diastolic LV dysfunction characterised by higher LV filling pressure [42]. On the other hand, the urinary collagen III fragment p107460 was associated with better diastolic LV function and lower LV filling pressure (Table 3 and Fig 2) and lower levels of TIMP-1 (Table 5). In patients with heart failure due to ischaemic heart disease or dilated cardiomyopathy [37], serum PIIINP levels are highly correlated with the myocardial collagen III volume fraction. The positive association between the urinary collagen I fragment p73697 and circulating PIIINP (Table 5), formed in a 1:2 stoichiometric ratio during the conversion of procollagen III to mature collagen III (S2 Fig), probably reflects the joint increase in both collagen subtypes [49] during myocardial fibrosis.

Strong points of our study are the availability of Doppler indexes of early subclinical diastolic LV dysfunction measured on a continuous scale, the application of two approaches in the statistical analysis, and the demonstration of a pathophysiologically plausible correlation between sequenced urinary collagen fragments and the serum biomarkers of collagen turnover. The epidemiological angle enhances the relevance of our findings over and beyond that of case-control studies involving selected heart failure patients, who represent the end stage of a long pathogenetic process confounded by multiple comorbidities and poly-medication. However, our present study must also be interpreted within the context of its limitations. First, our findings originate from a cross-sectional analysis and therefore reflect a snapshot in each individual participant. From this point of view our results should be considered as hypothesis generating. Whether or not, the urinary proteomic and serum biomarkers can predict the course over time of diastolic LV dysfunction remains to be confirmed in longitudinal studies. Second, the pathogenetic drivers leading to diastolic LV dysfunction are multifaceted each with different contributions among people at risk. Whether or not, the urinary collagen markers can predict the course over time of diastolic LV dysfunction remains to be proven in longitudinal studies. Third, we could not apply the simplified US criteria for the diagnosis of diastolic LV dysfunction in clinical practice (Fig 1) for the simple reason that they do not align with the gradation from normal to impaired diastolic function in the general population. However, our classification system passed expert peer review [7,9–11,18,21]. Finally, epidemiological studies demonstrate association and the causal interpretation of associations between traits of interest and biomarkers rests on a careful interpretation of the literature.

In conclusion, by sequencing urinary collagen I and III fragments and by linking diastolic LV function with urinary and serum collagen biomarkers, our current findings generalize previous observations in patients to the population at large. Our current observations support the concept of porting the use of multidimensional biomarkers measured on diverse platforms or in different media, e.g. urine and serum, to clinical practice to enable a personalised approach to the diagnosis, prevention and treatment of diastolic LV dysfunction, a high-risk condition [7] that affects 25% of the general population [6]. Furthermore, our current study highlights research tracks to be pursued in the future, such as showing parallelism between concomitant changes in diastolic LV function and biomarkers in longitudinal studies and proving concordance in the proteomic profiles of urine, serum or plasma and the myocardium.

## Supporting Information

S1 Table Glossary of echocardiographic measurement reflecting diastolic left ventricular function (DOC)

S2 Table List of urinary collagen fragments (DOC)

S3 Table Covariables selected by stepwise regression (DOC)

S4 Table Multivariable-adjusted associations of tissue Doppler indexes with urinary peptides (DOC)

S5 Table Urinary biomarkers by category of diastolic LV function (DOC)

S1 Fig –Log10(p) probability plot of the multivariable-adjusted associations of various indexes of diastolic left ventricular function with the urinary peptides (DOC)

S2 Fig Circulating biomarkers of collagen turnover (DOC)
