# Supplementary material for: Diastolic Left Ventricular Function in Relation to Urinary and Serum Collagen Biomarkers in a General Population
Source: PLoS One. 2016 Dec 13;11(12):e0167582. doi: 10.1371/journal.pone.0167582 (PMC5154519; doi:10.1371/journal.pone.0167582)
Supplement: S1 Table — (DOC) [file pone.0167582.s001.doc]

**S1 Table.**

**Glossary of echocardiographic measurement reflecting diastolic left ventricular function**

| Measurement | Description | Interpretation |
| --- | --- | --- |
| E, A, E/A  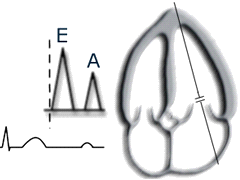 | Peak velocities of the transmitral blood flow, as measured by standard Doppler techniques during rapid LV filling in early diastole (E) and during atrial contraction in late diastole (A) | E (cm/s) decreases with impaired relaxation during the early stage of diastolic LV dysfunction, but increases in the later stages with onset of LV stiffening. During progression from mild to severe diastolic LV dysfunction, the A wave (cm/s) changes in directions opposite to E. The E/A ratio is decreased in early diastolic LV dysfunction. Because mitral inflow patterns are highly sensitive to preload (left atrial pressure) and change as diastolic dysfunction progresses, the use of mitral valve inflow patterns to assess diastolic function remains limited.  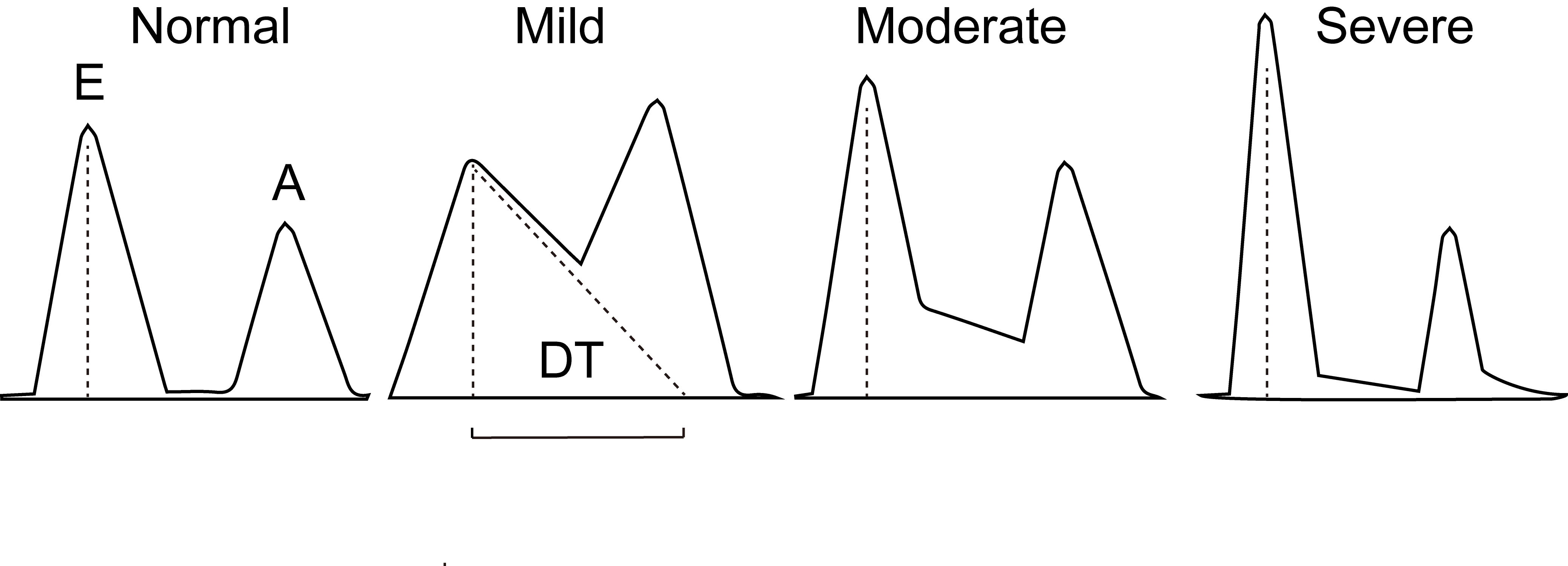 |
| e’, a’, e’/a’  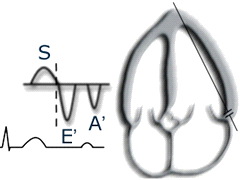 | Peak velocities of the mitral annular movement, as measured by tissue Doppler imaging (TDI) during rapid LV filling in early diastole (e’) and during atrial contraction in late diastole (a’) | e’ (cm/s) progressively decreases during the progression of diastolic LV dysfunction. a’ (cm/s) increases in the early stage of diastolic LV dysfunction, but thereafter decreases with advancing disease. TDI assessment of diastolic LV function is less load dependent than that provided by standard Doppler techniques. e’ is resistant to changes in LV filling pressure. The e’/a’ ratio decreases in early diastolic LV dysfunction, but increases with further progression.  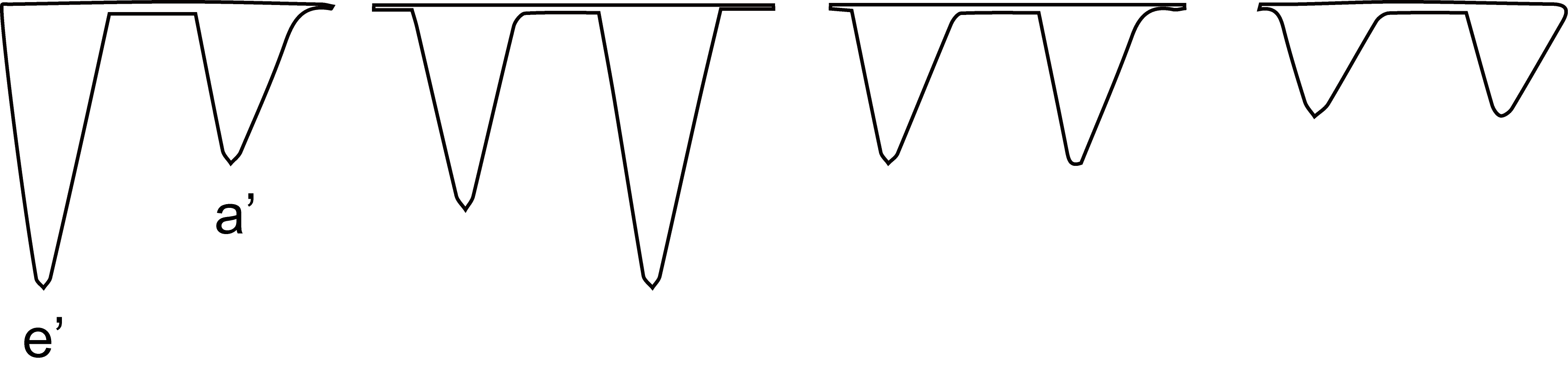 |
| E/e’ | Transmitral E / mitral annular e’ | This ratio reflects LV filling pressure and increases during progression of diastolic LV dysfunction. |
| Ad, ARd, LAVI | Duration of the transmitral A wave and of the reversal of flow in the pulmonary veins; left atrial volume normalised to body surface area | Ad < ARd + 10 (ms) and LAVI ≥ 28 mL/m^2^ confirm that LV filling pressure is high. |
| DT, IVRT | Deceleration time and isovolumetric relaxation time | DT (ms) and IVRT (ms) initially lengthen as LV relaxation is impaired, but shorten with more severe LV dysfunction. |
