# Supplementary material for: Diastolic Left Ventricular Function in Relation to Urinary and Serum Collagen Biomarkers in a General Population
Source: PLoS One. 2016 Dec 13;11(12):e0167582. doi: 10.1371/journal.pone.0167582 (PMC5154519; doi:10.1371/journal.pone.0167582)
Supplement: S2 Table — (DOC) [file pone.0167582.s002.doc]

**S2 Table.**

**Urinary collagen fragments with known amino-acid sequence (Starts)**

| ID | Sequence | Protein |
| --- | --- | --- |
| 4976 | DpGKNGDKG | Collagen alpha-2(I) chain |
| 13342 | ApGDKGESGPS | Collagen alpha-1(I) chain |
| 14906 | DGRpGPpGPpG | Collagen alpha-1(I) chain |
| 17694 | ApGDRGEpGpP | Collagen alpha-1(I) chain |
| 26113 | GppGPDGNKGEpG | Collagen alpha-2(I) chain |
| 27350 | DKGETGEQGDRG | Collagen alpha-1(I) chain |
| 32171 | ApGDRGEpGPpGPA | Collagen alpha-1(I) chain |
| 35339 | ApGDRGEpGPpGPAG | Collagen alpha-1(I) chain |
| 37903 | GPpGPpGPpGPpGPPS | Collagen alpha-1(I) chain |
| 37949 | KGDKGAMGEpGPPGp | Collagen alpha-3(IV) chain |
| 38605 | SpGSPGPDGKTGPpGP | Collagen alpha-1(I) chain |
| 40243 | SpGSpGPDGKTGPPGp | Collagen alpha-1(I) chain |
| 42304 | DGQpGAKGEpGDAGAK | Collagen alpha-1(I) chain |
| 43442 | VGPpGPpGPpGPPGPPS | Collagen alpha-1(I) chain |
| 43543 | GSpGSpGPDGKTGPPGp | Collagen alpha-1(I) chain |
| 44618 | VGPpGPpGPpGpPGPPS | Collagen alpha-1(I) chain |
| 48106 | SpGSpGPDGKTGPPGpAG | Collagen alpha-1(I) chain |
| 50840 | DGApGKNGERGGpGGpGP | Collagen alpha-1(III) chain |
| 52189 | pGKpGEDGEpGRNGNp | Collagen alpha-2(V) chain |
| 53035 | VGPpGPpGPpGPpGPPSAG | Collagen alpha-1(I) chain |
| 54525 | GLpGTGGPpGENGKpGEp | Collagen alpha-1(III) chain |
| 55582 | NGApGNDGAKGDAGApGApG | Collagen alpha-1(I) chain |
| 55756 | EpGSpGENGApGQmGPR | Collagen alpha-1(I) chain |
| 57531 | TGSpGSpGPDGKTGPPGpAG | Collagen alpha-1(I) chain |

**S2 Table.**

**Urinary collagen fragments with known amino-acid sequence (Continued)**

| ID | Sequence | Protein |
| --- | --- | --- |
| 57537 | NDGApGKNGERGGpGGpGP | Collagen alpha-1(III) chain |
| 60149 | GNDGApGKNGERGGpGGpGP | Collagen alpha-1(III) chain |
| 61332 | ApGAPGGKGDAGApGERGPpG | Collagen alpha-1(III) chain |
| 69769 | DGESGRPGRpGERGLpGPpG | Collagen alpha-1(III) chain |
| 70413 | DGESGRpGRpGERGLpGPpG | Collagen alpha-1(III) chain |
| 70635 | NSGEpGApGSKGDTGAKGEpGP | Collagen alpha-1(I) chain |
| 70674 | EGSpGRDGSpGAKGDRGETGP | Collagen alpha-1(I) chain |
| 71602 | PpGEAGKpGEQGVpGDLGAPGP | Collagen alpha-1(I) chain |
| 72596 | NGDDGEAGKPGRPGERGPpGp | Collagen alpha-1(I) chain |
| 72641 | QGLQGQQGGAGpTGpPGEpGDP | Collagen alpha-2(V) chain |
| 72896 | SGEpGApGSKGDTGAKGEpGPVG | Collagen alpha-1(I) chain |
| 73177 | DAGApGAPGGKGDAGApGERGPpG | Collagen alpha-1(III) chain |
| 73246 | NGDDGEAGKpGRpGERGPpGP | Collagen alpha-1(I) chain |
| 73697 | GNSGEpGApGSKGDTGAKGEPGp | Collagen alpha-1(I) chain |
| 74065 | DAGApGApGGKGDAGApGERGPpG | Collagen alpha-1(III) chain |
| 75846 | GPpGEAGKpGEQGVpGDLGApGP | Collagen alpha-1(I) chain |
| 77018 | DGQPGAKGEpGDAGAKGDAGPPGp | Collagen alpha-1(I) chain |
| 77763 | DGQpGAKGEpGDAGAKGDAGPPGp | Collagen alpha-1(I) chain |
| 77952 | VGEpGPAGSKGESGNKGEpGSAGP | Collagen alpha-2(I) chain |
| 78332 | AGPpGEAGkPGEQGVPGDLGAPGp | Collagen alpha-1(I) chain |
| 78843 | NSGEpGApGSKGDTGAKGEpGPVG | Collagen alpha-1(I) chain |
| 79136 | AGPpGEAGKpGEQGVpGDLGApGP | Collagen alpha-1(I) chain |
| 79626 | NSGEpGApGSKGDTGAkGEpGPVG | Collagen alpha-1(I) chain |
| 80891 | ADGQPGAKGEpGDAGAKGDAGPPGp | Collagen alpha-1(I) chain |
| 81196 | NGApGNDGAKGDAGApGApGSQGApG | Collagen alpha-1(I) chain |

**S2 Table.**

**Urinary collagen fragments with known amino-acid sequence (Continued)**

| ID | Sequence | Protein |
| --- | --- | --- |
| 81758 | ADGQpGAKGEpGDAGAKGDAGPpGP | Collagen alpha-1(I) chain |
| 84542 | QNGEpGGKGERGAPGEKGEGGppG | Collagen alpha-1(III) chain |
| 85315 | ANGApGNDGAKGDAGApGApGSQGApG | Collagen alpha-1(I) chain |
| 85761 | ADGQpGAKGEpGDAGAKGDAGPpGPA | Collagen alpha-1(I) chain |
| 89233 | KGNSGEpGApGSKGDTGAKGEpGPVG | Collagen alpha-1(I) chain |
| 90344 | GKNGDDGEAGKpGRpGERGPpGPQ | Collagen alpha-1(I) chain |
| 91542 | LDGAKGDAGPAGPKGEpGSpGENGApG | Collagen alpha-1(I) chain |
| 98660 | GApGQNGEpGGKGERGApGEKGEGGPpG | Collagen alpha-1(III) chain |
| 99577 | PGRpGLDGERGRPGPAGPpGPpGPSSN | Collagen alpha-6(IV) chain |
| 99808 | LTGPIGPPGpAGApGDKGESGPSGPAGPTG | Collagen alpha-1(I) chain |
| 104786 | pGMPGADGPPGHPGKEGppGEKGGQGpPG | Collagen alpha-1(V) chain |
| 105352 | SGHPGSPGSPGYQGPpGEPGQAGPSGPpGP | Collagen alpha-1(III) chain |
| 107460 | KNGETGPQGPPGPTGPGGDKGDTGPpGpQG | Collagen alpha-1(III) chain |
| 111001 | ERGEAGIpGVpGAKGEDGKDGSpGEpGANG | Collagen alpha-1(III) chain |
| 112106 | PQGPpGPTGpGGDKGDTGPpGPQGLQGLpGT | Collagen alpha-1(III) chain |
| 115491 | ESGREGApGAEGSpGRDGSpGAKGDRGETGP | Collagen alpha-1(I) chain |
| 118163 | LTGSpGSpGpDGKTGPPGPAGQDGRPGPpGppG | Collagen alpha-1(I) chain |
| 119538 | GpSGASGERGPpGPMGpPGLAGPPGESGREGAPG | Collagen alpha-1(I) chain |
| 125402 | PpGESGREGApGAEGSpGRDGSpGAKGDRGETGP | Collagen alpha-1(I) chain |
| 133430 | PpGPSGpRGQPGVMGFpGPKGNDGAPGKNGERGGPGG | Collagen alpha-1(III) chain |
| 134470 | PpGADGQpGAKGEPGDAGAKGDAGPPGpAGPAGPPGpIGN | Collagen alpha-1(I) chain |
