# Supplementary material for: Diastolic Left Ventricular Function in Relation to Urinary and Serum Collagen Biomarkers in a General Population
Source: PLoS One. 2016 Dec 13;11(12):e0167582. doi: 10.1371/journal.pone.0167582 (PMC5154519; doi:10.1371/journal.pone.0167582)
Supplement: S3 Table — (DOC) [file pone.0167582.s003.doc]

**S3 Table.**

**Covariables selected by stepwise regression**

| Covariables |  | E peak | A peak | E/A | e’ | a’ | e’/a’ | E/e’ |
| --- | --- | --- | --- | --- | --- | --- | --- | --- |
| R^2^ |  | 0.27 | 0.58 | 0.66 | 0.75 | 0.49 | 0.73 | 0.46 |
| Female sex (0,1) |  | 8.66§ | 5.82§ | 0.05† | … | –0.88§ | 0.09‡ | 1.11§ |
| Age (+15.5 yrs) |  | –6.56§ | 10.0§ | –0.33§ | –2.41§ | 1.26§ | –0.46§ | 0.80§ |
| Body mass index (+4.2 kg/m^2^) |  | … | 2.82§ | –0.07§ | –0.65§ | 0.35§ | –0.14§ | 0.30§ |
| Mean arterial pressure (+10.7 mm Hg ) |  | … | 2.14§ | –0.02† | –0.32§ | … | –0.03† | 0.32§ |
| Heart rate (+9.7 bpm) |  | –3.15§ | 3.62§ | –0.14§ | –0.25§ | 0.68§ | –0.12§ | –0.12* |
| Serum creatinine (+15.4 μmol/L) |  | … | … | 0.02* | 0.14† | … | … | … |
| Total cholesterol (+0.97 mmol/L) |  | … | … | –0.03‡ | –0.19‡ | 0.16‡ | –0.06§ | … |
| Log γ−glutamyltransferase (× 2) |  | –0.96* | –0.76* | … | –0.26‡ | 0.15† | –0.04† | … |
| Fasting plasma glucose (+0.79 mmol/L) |  | … | … | … | … | … | … | 0.09* |
| LVMI (+21.9 g/m^2^) |  | … | … | … | –0.27§ | –0.20‡ | … | 0.32§ |
| On treatment with diuretics (0,1) |  | … | … | … | –0.41* | –0.45† | … | 0.51† |
| On treatment with β-blockers (0,1) |  | … | 1.94* | –0.07† | –0.60‡ | –0.75§ | … | 0.41† |
| On treatment with RAAS inhibitors (0,1) |  | –3.40* | … | … | … | … | … | –0.39* |

The variables considered for entry into the models included sex, age, body mass index, mean arterial pressure, heart rate, serum total cholesterol, γ‑glutamyltransferase (as index of alcohol intake) and creatinine, fasting plasma glucose, LVMI, and treatment with diuretics (thiazides, loop diuretics and aldosterone antagonists), β‑ blockers, inhibitors of the renin-angiotensin system (angiotensin-converting enzyme inhibitors or angiotensin type‑1 receptor blockers), and vasodilators (calcium channel blockers and α‑blockers). *P*‑values for variables to enter and stay in the regression models were set at 0.15. Significance of the association: * 0.05<*p*≤0.15; † *p*≤ 0.05; ‡ *p*≤ 0.01; § *p*≤ 0.001.
