# Supplementary material for: Diastolic Left Ventricular Function in Relation to Urinary and Serum Collagen Biomarkers in a General Population
Source: PLoS One. 2016 Dec 13;11(12):e0167582. doi: 10.1371/journal.pone.0167582 (PMC5154519; doi:10.1371/journal.pone.0167582)
Supplement: S4 Table — (DOC) [file pone.0167582.s004.doc]

**S4 Table.**

**Multivariable-adjusted associations of tissue Doppler indexes with urinary peptides**

| Urinary peptides (SD) | Collagen Type | Estimate (95% CI) | *p* |
| --- | --- | --- | --- |
| A peak |  |  |  |
| p107460 (863) | III | –1.404 (–2.477 to –0.330) | 0.0044 |
| p112106 (3149) | III | –1.291 (–2.361 to –0.221) | 0.011 |
| e’ peak |  |  |  |
| p70635 (728) | I | –0.149 (–0.342 to –0.007) | 0.038 |
| a’ peak |  |  |  |
| p72896 (389) | I | –0.203 (–0.343 to –0.064) | 0.0012 |
| p73697 (521) | I | –0.163 (–0.288 to –0.010) | 0.031 |
| e’/a’ peak |  |  |  |
| p77952 (1518) | I | –0.035 (–0.067 to –0.002) | 0.032 |
| E/e’ |  |  |  |
| p72896 (289) | I | 0.140 (–0.010 to 0.289) | 0.076 |
| p77018 (1504) | I | 0.211 (0.066 to 0.357) | 0.0012 |
| p107460 (863) | III | –0.165 (–0.316 to –0.014) | 0.026 |
| p115491 (2362) | I | 0.162 (0.016 to 0.308) | 0.022 |

Abbreviations: CI, confidence interval. We excluded 25 participants with proteinuria. All estimates were adjusted for sex, age, body mass index, mean arterial pressure, heart rate, serum total cholesterol, γ−glutamyltransferase and creatinine, plasma glucose, LVMI and treatment with diuretics, β−blockers and inhibitors of the renin-angiotensin system. Estimates express the change in the dependent variable for 1‑SD increase (given between parentheses) in the urinary peptide. *P*‑values are Bonferroni adjusted.
