# Supplementary material for: Diastolic Left Ventricular Function in Relation to Urinary and Serum Collagen Biomarkers in a General Population
Source: PLoS One. 2016 Dec 13;11(12):e0167582. doi: 10.1371/journal.pone.0167582 (PMC5154519; doi:10.1371/journal.pone.0167582)
Supplement: S5 Table — (DOC) [file pone.0167582.s005.doc]

**S5 Table.**

**Urinary biomarkers by category of diastolic LV function**

| Biomarkers |  | Normal (n = 587) | Dysfunction (n = 170) | *p* |
| --- | --- | --- | --- | --- |
| p77018 (I) |  | 2765 (6) | 3046 (122) | 0.048 |
| p107460 (III) |  | 1774 (35) | 1599 (69) | 0.029 |
| p77018/p107460 (I/III) |  | 2.25 (0.19) | 3.66 (0.38) | 0.0015 |

We excluded 25 participants with proteinuria. Values are arithmetic mean (SE). Adjustments included body mass index, serum total cholesterol, γ−glutamyltransferase and creatinine, plasma glucose, and treatment with diuretics, β−blockers and inhibitors of the renin-angiotensin system.
