# Supplementary material for: Diastolic Left Ventricular Function in Relation to Urinary and Serum Collagen Biomarkers in a General Population
Source: PLoS One. 2016 Dec 13;11(12):e0167582. doi: 10.1371/journal.pone.0167582 (PMC5154519; doi:10.1371/journal.pone.0167582)
Supplement: S1 Fig — (DOC) [file pone.0167582.s006.doc]

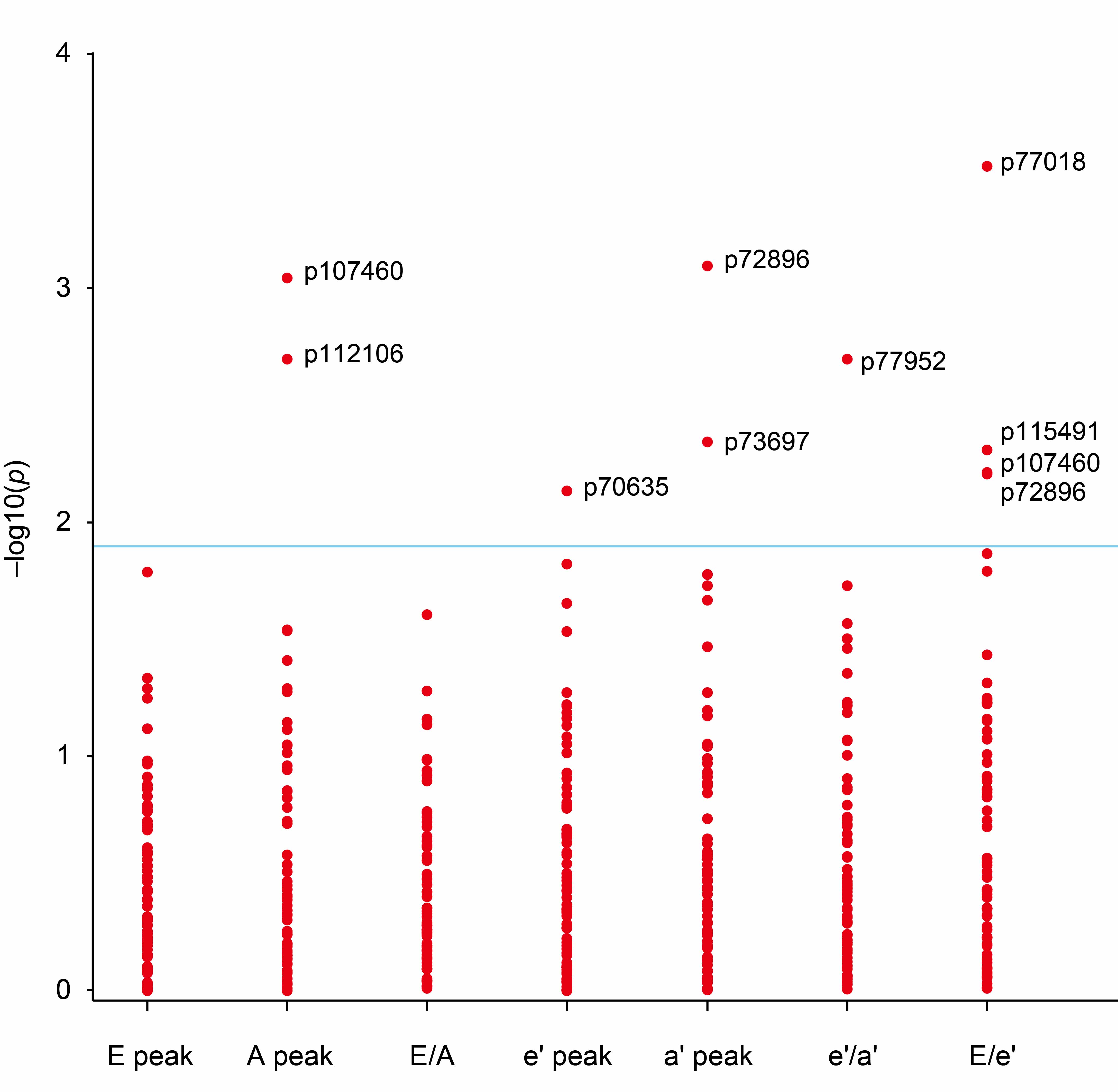


**S1 Fig.**

–Log10(*p*) probability plot of the multivariable-adjusted associations of various indexes of diastolic left ventricular function with the urinary peptides. The adjustment accounted for sex, age, body mass index, mean artery pressure, heart rate, serum total cholesterol, γ−glutamyltransferase and creatinine, fasting plasma glucose, LVMI, treatment with diuretics, β−blockers and inhibitors of the renin-angiotensin system. The horizontal line denotes the significance level with Bonferroni correction applied.
