# Supplementary material for: Diastolic Left Ventricular Function in Relation to Urinary and Serum Collagen Biomarkers in a General Population
Source: PLoS One. 2016 Dec 13;11(12):e0167582. doi: 10.1371/journal.pone.0167582 (PMC5154519; doi:10.1371/journal.pone.0167582)
Supplement: S2 Fig — (DOC) [file pone.0167582.s007.doc]

**
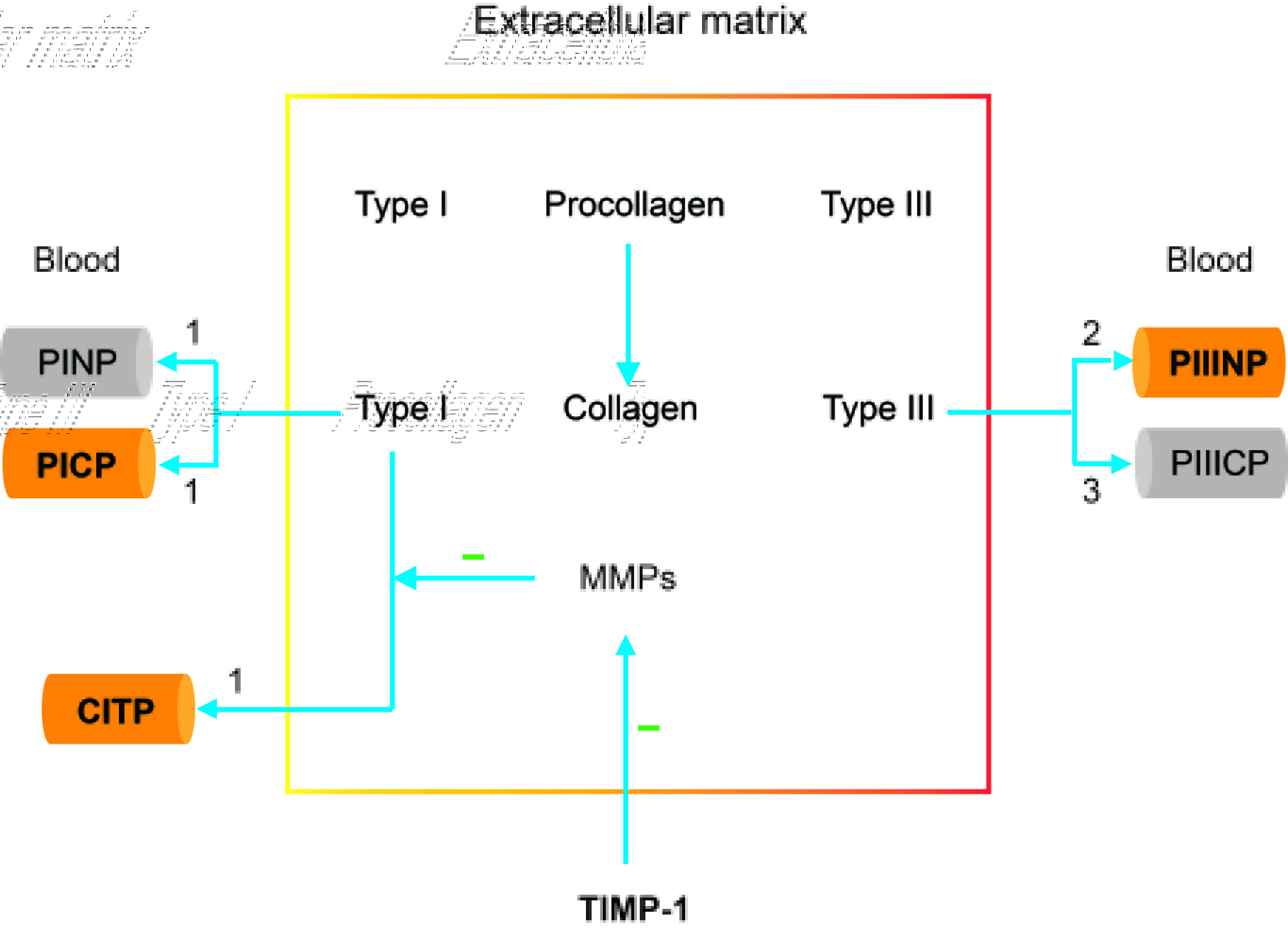
**

**S2 Fig.**

Circulating biomarkers of collagen turnover. PINP and PICP are released during conversion of procollagen I to collagen I, and CITP during the degradation of collagen I by matrix metalloproteinases, which are inhibited by TIMP‑1. PIIINP and PIIICP are released during conversion of procollagen III to collagen III. Numbers alongside arrows indicate the stoichiometric ratio. In the present study, serum levels of PICP, CITP, PIIINP and TIMP‑1 were measured. PICP is a direct indicator of collagen I synthesis and CITP of collagen I degradation. PIIINP is an imperfect indicator of collagen III synthesis, because PIIINP remains partially bound to the surface of collagen III fibres and is therefore also released during breakdown of these fibres (JACC 2015; 65: 2449–2456). Abbreviations: PICP, procollagen I carboxy-terminal propeptide; PINP, procollagen I amino-terminal propeptide; CITP, carboxyterminal telopeptide of collagen I; MMPs, matrix metalloproteinases; TIMP‑1, tissue inhibitor of the matrix metalloproteinase type 1; PIIICP, procollagen III carboxy-terminal propeptide; and PIIINP, procollagen III amino-terminal propeptide.
